# Response and Tolerance Mechanism of Cotton *Gossypium hirsutum* L. to Elevated Temperature Stress: A Review

**DOI:** 10.3389/fpls.2016.00937

**Published:** 2016-06-30

**Authors:** Kashif Rafiq Zahid, Farhan Ali, Farooq Shah, Muhammad Younas, Tariq Shah, Durri Shahwar, Waseem Hassan, Zahoor Ahmad, Chao Qi, Yanli Lu, Amjad Iqbal, Wei Wu

**Affiliations:** ^1^Hubei Key Laboratory of Genetic Regulation and Integrative Biology, Central China Normal UniversityWuhan, China; ^2^Cereal Crops Research InstitutePirsabak, Nowshera, Pakistan; ^3^Department of Agriculture, Abdul Wali Khan University MardanKhyber Pakhtunkhwa, Pakistan; ^4^Department of Biotechnology, Mohi-ud-Din Islamic UniversityAzad Jammu and Kashmir, Pakistan; ^5^Department of Agricultural Economics and Management, Huazhong Agricultural UniversityWuhan, China; ^6^Department of Plant Breeding and Genetics, University of SwabiKhyber Pakhtunkhwa, Pakistan; ^7^Department of Soil and Environmental Sciences, Muhammad Nawaz Shareef University of AgricultureMultan, Pakistan; ^8^Key Lab of Crop Disease Monitoring and Safety Control, Huazhong Agricultural UniversityWuhan, China; ^9^Maize Research Institute, Sichuan Agricultural UniversityWenjian Sichuan, China; ^10^College of Agronomy, Northwest A&F UniversityYangling, China

**Keywords:** cotton, heat stress, signal transduction, abscisic acid

## Abstract

Cotton is an important multipurpose crop which is highly sensitive to both biotic and abiotic stresses. Proper management of this cash crop requires systematic understanding of various environmental conditions that are vital to yield and quality. High temperature stress can severely affect the viability of pollens and anther indehiscence, which leads to significant yield losses. Cotton can respond to withstand adverse environmental condition in several phases among which the accumulation of chemicals is extremely vital. Calcium, kinases, reactive oxygen species, carbohydrate, transcription factors, gene expression regulation, and plant hormones signaling pathways are playing a handy role in activating the major genes responsible to encounter and defend elevated temperature stress. The production of heat shock proteins is up-regulated when crops are unleashed to high temperature stress. Molecular breeding can play a functional role to identify superior genes for all the important attributes as well as provide breeder ready markers for developing ideotypes. The development of high-temperature resistant transgenic cultivars of cotton can grant a stability benefit and can also ameliorate the production capacity in response to elevated temperature.

## Introduction

Upland cotton (*Gossypium hirsutum* L.) is one of the most important cash crops that provide fiber to the textile industries around the world. According to the rough estimation regarding the world production of cotton, 80% comes from Brazil, China, India, Pakistan, Turkey, USA, and Uzbekistan. This crop contributes a major portion to the gross national product (GNP) of many countries. The accumulation of greenhouse gasses along with carbon dioxide in the atmosphere at soaring rate might offer threats to agriculture in near future. It has been mentioned that global warming (GW) will result in more environmental fluctuations in majority of agricultural zones of the globe (Solomon, 2007). The increase of 0.6°C in temperature, during 1990–2000 can be attributed to GW (Tebaldi et al., 2007). The constant emissions of GHGs (in terms of frequency, intensity, and duration of radiations) will bring great changes in annual average temperatures (2.5–4.3°C) in the main crop-growing areas of the world by 2080 (Tebaldi et al., 2007). A harmful effect of non-living factors on the living organism is known as abiotic stress. As plants are sessile, they have to withstand several stresses during their life period for growth, survival and continuity of generation. Among these abiotic stresses the most serious threat for global food security is high temperature. It is proven that for each degree (Celsius) rise in temperature can affect the crop yield up to 17% in a growing season (Lobell and Asner, 2003). Such changes in the temperature can bring irregular pattern of rain and drought that can lead to high losses in revenue (for example it cost United States, ∼ US$ 4.2 billion in agricultural losses during the month of August 2000; Mittler, 2006). Thus it is not difficult to quantify the fast and harsh effect of high temperatures on the reduction of agricultural production.

China, Pakistan, India, and Sudan have the ability to produce cotton even at slightly higher temperatures (**Table 1**) than the current averages and thus covering almost 75% of the world cotton area (ICAC, 2009). If the mean annual temperature of the globe increases at such rate then the leading producers might experience great losses in yield. Conversely, those areas which are already producing cotton at temperatures close to 40°C would be affected by adverse climatic conditions resulting dramatic losses in per unit production. It is obvious that elevated temperature is among the main hurdles in crop progression and productivity, which is why it has a detrimental effect on crop production. The blossom days and growth of cotton reduce with high temperature. Heat stress during late July and the beginning of August can lead to the stunted vegetative growth, reduced boll size, and number of seeds/boll. This review not only highlights the current research findings regarding responses of cotton crop to high temperature, but also focuses on the genotypic variation and morphological characteristics conferring tolerance. Strategies in order to mitigate the heat stress are the main focus with emphasis on certain key elements providing tolerance. The role of miRNAs and abscisic acid (ABA) in signal transduction and the process of methylation helping the crop to withstand adverse environmental conditions are also described.

**Table 1:** Monthly average maximum temperature (in °C) for a 6-months cotton season crop in some major cotton producing countries.

Country	Months						Mean
	1	2	3	4	5	6	
Turkey	20	27	33	38	38	33	31.5
China	21	27	32	31	30	27	28.2
US	24	28	32	34	33	30	30.2
Australia	27	30	33	34	33	31	31.5
Argentina	30	33	34	34	32	32	32.5
India	36	41	40	40	36	36	37.5
Pakistan	40	40	37	37	35	33	36.5

*The table is reprinted with permission from ICAC (2009)*.

## Relation of High Temperature to Development and Growth

Photoperiod and temperature are the fundamental factors of crops at all stages of development and reproduction. The most advantageous temperature ranges from 20 to 30°C for cotton (Reddy et al., 1996). The special effects of high temperature on different developmental stages like germination, growth of seedling, vegetative production, morphological development, and maturity attributes are very important. Cotton is extremely sensitive to temperature throughout all developmental stages. However, the reproductive stage is particularly susceptible to high temperature during flowering time (Oosterhuis, 2002). The impact of high temperature on pollen tube germination, growth and elongation shows that temperature above (28–30°C) adversely affects cotton reproductive performance. It has been observed that the pollen germination was sharp when temperature was maintained at 28°C (Burke et al., 2004). Germination rate decreased as temperatures increases above (28°C) and declined abruptly at temperatures above 37°C.

### Effect of High Temperature on Root Growth

Genotypic differences in cotton during seed germination and root development under severe temperatures are crucial. It is therefore, needed to grow the crop in specific time period to escape adverse environmental conditions. The optimum day temperature for root development is 22/30, while the night temperature should be 27/35°C. The temperature beyond 32/40°C distorted the distribution and growth of roots. This temperature also causes shorter and stunted roots even under optimum nutrients and ideal water conditions (Reddy et al., 1997). Furthermore, literature demonstrated that heat patience seedlings are vital in nearly all dry land cotton producing area (Burke, 2001). Therefore, it is imperative to keep in view the optimum ideal moisture level during sowing. If the wind velocity and soil temperature are high at sowing time it will cause rapid loss of moisture. These circumstances adversely affect the development of root system and final production. Temperature has been reported to influence the fatty acid composition in seeds, roots, and leaves. Heat stress is the main cause of numerous changes in living cells that ultimately distress membrane structure and functions (Tsvetkova et al., 2002). Genetic differences in rooting system are directly related to plant productivity and primarily increases root branching and distribution. Strong rooting system can boost cotton yield under drying soil profile conditions. Meanwhile, the enhancement of vascular system can increase lateral root production, which provides strength against abiotic stresses. The genotypes that have deep roots with enlarged lateral root system are more resilient to abiotic stress (Cook and El-Zik, 1992). Therefore, it would be ideal to find out genes for this important trait and transform it the crop to a high yielding genotypes. Another approach can be simple breeding and crosses of different genotypes to create variation for root deployment to withstand severe environmental condition.

### Elevated Temperature Stress Suppresses Photosynthesis

Photosynthesis is the main factor of crop growth and cotton is highly sensitive to temperature for maintaining food reserve. Photosynthetic rates are usually high at about 30°C but significantly declines with each additional degree increase in temperature. The high day temperature during vegetative growth is one of the severe sources for yield reduction and affecting the morphological attributes leading toward stunted growth. Photosynthesis is also expected to decrease as temperature increases (Schuster and Monson, 1990) resulting in shorter plants and ultimately loss of boll production. The deactivation of Rubisco at high temperatures protects the PSII. This deactivation was proposed to be the principal restraint of photosynthesis at 40°C (Crafts-Brandner and Law, 2000). Barua et al. (2003) have shown a close link between heat shock proteins (HSPs) and crop’s photosynthetic capability. The photosynthetic electron flow of leaves can be disturbed initially upon the exposure to even heat stress of moderate level (Schrader et al., 2004). However, such effect of adverse temperature on plant photosynthesis is non-reversible due to the inhibition of electron flow (Schrader et al., 2004). The effects of reasonable heat stress on thylakoid reactions are effortlessly missed in spite of physiological significance. The structural changes in thylakoid membrane owing to heat stress were accomplished by freeze fracture and these changes may be the basis of changes in thylakoid function (Gounaris et al., 1984). The property of these thylakoid membranes against high temperature was also sustained by the findings that mutants are more tolerant to heat stress lacking trienoic fatty acids (Murakami et al., 2000). Therefore, the injury to thylakoid because of modest heat stress is not resultant of damage to PSII but because it involves pathways associated to cyclic electron flow and conceivably the cytochrome complex.

### The Impact of High Temperature on Flowering, Vegetative, and Fruiting Branches

**FIGURE 1:**
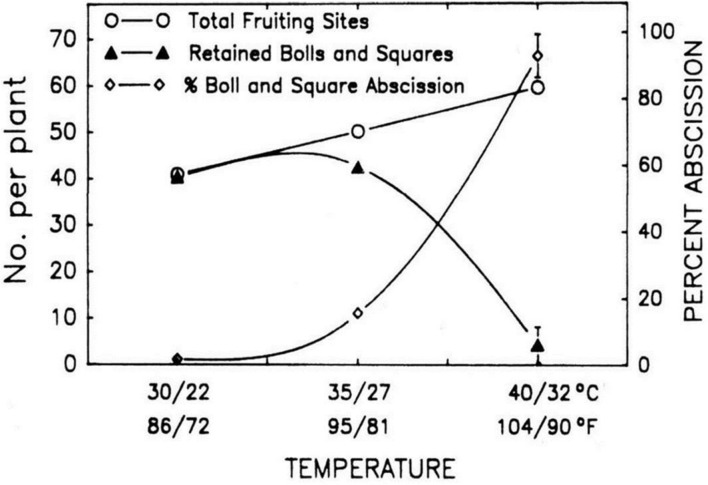
**The Impact of increment in day/night temperature on fruiting sites, bolls and squares**. The figure is reprinted with permission from Reddy et al. (1992b).

Leaf area is also extremely responsive to extreme temperature and the optimum temperature ranges from 26 to 28°C (Reddy et al., 1992a,b). Flowering branches can also be severely influenced by temperature (Reddy et al., 1997). Ehlig and LeMert (1973) found that flowering ratio decreased approximately 3 weeks after the periods when maximum temperature go beyond 42°C. Heat stress during flowering time caused dropping of squares and flowers when the temperature is increased above 30°C during day time. The number of seed and boll size drastically reduced when the average temperatures cross the limits of 28°C and extremely small number of fruit retain once the average temperatures enhanced above 32°C. The development of vegetative as well as fruiting branches can drastically be affected by high temperature (**Figure 1**). Fruiting sites are observed to increase by 50% when temperature is raised by 10°C, i.e., from 30 to 40°C. However, bolls are reduced spectacularly above 35°C and approaches to zero when the temperature is above 40°C (Reddy et al., 1997). It is stated that newly developed bolls usually shed when mean day temperature is either 32°C or even high.

### Elevated Temperature Stress Leads to Deficient Reproduction

Cotton crop starts flowering approximately after 8 weeks following plant emergence in an indeterminate growth habit (Oosterhuis, 1999). Warm night temperatures always results in poor reproductive performance. Fertilization occurs between 12 and 24 h once pollens are released because of slow development of pollen tube. High temperature injury during flowering can cause failure of either pollination or fertilization and even both, resulting in less number of bolls setting. Pollen grains are sensitive to extreme temperatures than ovule and can account for reduce fertilization under stress because of elevated temperature. Pollen tubes and pollen grains require high amount of energy relative to other vegetative tissues (Burke et al., 2004). Heat stress restricts the ability of source and restrains the formation of carbohydrate and its distribution for developing sinks leads toward low production (Liu et al., 2006). This process also reduces the photosynthetic ability; boost the dark respiration and photorespiration, and slow down translocation. Heat stress also reduces the level of carbohydrates in the grains containing pollen and the amount of adenosine triphosphate in the stigmatic tissue (Snider et al., 2009). Extreme temperature limits fertilization efficiency by disturbing carbohydrate metabolism and levels of calcium. Furthermore, boosting oxidative hassle in reproductive tissues during the reproductive phase of cotton can decrease the yield to a considerable amount.

### Elevated Temperature Stress Leads to Pollen Infertility

**FIGURE 2:**
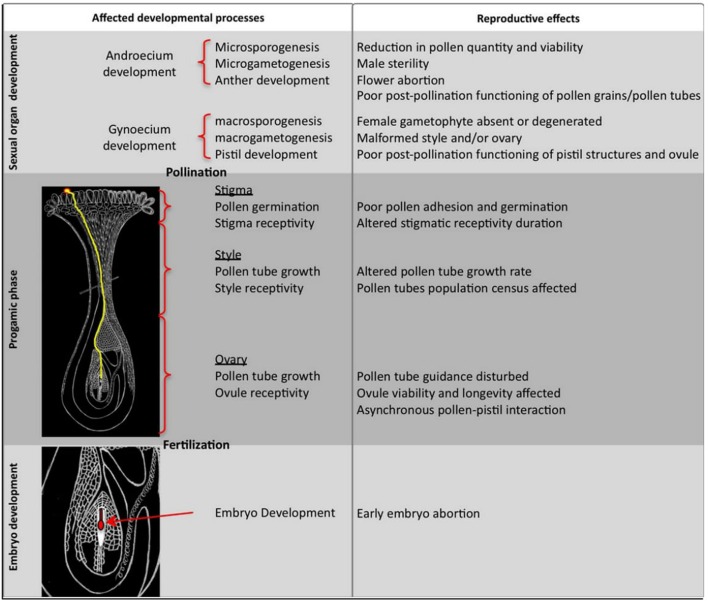
**Impact of heat stress on pollen development and function during the sexual reproductive phase**. The figure is reprinted with permission from Hedhly (2011).

During high temperature stress pollen grains of cotton lose their ability of fertilization. Pollen grains are highly sensitive to heat stress during its entire life period. The length of the pollen tubes is also sensitive to temperature. Tube lengths decreased drastically once temperature reached 34°C and approached to zero at 43°C. To understand the mechanism of anther abortion during high temperature stress in cotton, *GhCKI* gene need to be study in depth that has been over-expressed in cotton (Min et al., 2013). The expression of *GhCKI* was found high in the reproductive organs mainly in the well-developed anthers (Min et al., 2013). *GhCKI* could be induced under high temperature in anthers of high temperature-sensitive lines. This idea particularly highlighted that such kind of genes are crucial during stress and we need to find out all resistant genes in cotton for manipulating the germplasm in desirable direction. During high temperaturestress several genes induce anther indehiscence, while out of 88 genes, 5 genes can control the metabolism of carbohydrates and cells death (Min et al., 2013). Numerous pathways have been discovered that causes male sterility when plant gets exposed to severe temperatures (Zhang et al., 2010). The impact of heat shock on pollen functionality and further development during the sexual reproductive phase has been observed (**Figure 2**). Enzymes which are involved in the metabolism of carbohydrates and transport are particularly important during stresses. These enzymes can be used as indicators of losses in pollen viability due to temperature fluctuations. Male sterility comes from anther indehiscence during heat stress. Recently, experiment has been performed to determine the cause of male sterility by over-expressing *GhCK1* gene in cotton and *Arabidopsis*. The cross sections of anthers from transgenic plants have been observed and further classified according to the well-established system at various stages of anther development (Sanders et al., 1999). On the basis of anther morphology and characteristics, different anthers have been observed during stages 10–14 (Min et al., 2013). It has been found that the tapetum in wild-type plants was shortened during the 10th stage of development, while the juvenile microspores were swollen (Min et al., 2013). However, by the 11th stage the tapetum is entirely degenerated leading to developed pollen grains at the 12th, finally the anthers open up releasing the mature pollen grains. These findings showed that once the *GhCKI* protein accumulates, it stops degeneration of tapetal that might leads to anther’s indehiscence (Min et al., 2013).

### Elevated Temperature Stress Reduces Quantity of Pollens

The quantity of pollen grains applied to a stigma, controls both the growth of pollen tubes and subsequently the progeny. The first aspect is the reduction in the rate of pollen tube development due to low number of pollen grains and the second, yet major aspect is the influence of limited pollination upon the progeny. In each case, variation among the resultant plants was high when pollen quantity was lower than normal. To demonstrate the relationship between the speed of pollen tube growth rates and the quantity of pollen following experiment has been performed. In an experiment by Ter-Avanesian (1978), 20 pollen grains were applied on the stigmas of emasculated flowers of cotton; in a second experiment, an unlimited quantity of pollen grains was shattered on the stigmas of emasculated flowers. Pollen tubes from limited quantity of pollens reached the ovule in 15 h while those from unlimited quantity of pollens required 8 h (**Table 2**). The growth rate of pollen tubes were nearly two times faster in the latter case. The slow growth rate of tubes due to limited quantity of pollen grains can be explained by the disturbance of the physiological interaction of pollen grains and stigma. Those cultivars which possessed long anthers are resistant to heat stress at flowering because of high number of grains with pollen/anther.

**Table 2:** The impact of number of pollen grains upon variation in cotton (average values).

Pollen quantity	No. of plants	Yield/plant(g)	Boll no./ plant	Plant height (cm)
20 grains	5	175.28 (48.06)	33.58 (44.6)	105.0 (24.3)
100 grains	6	164.58 (42.5)	33.53 (28.9)	103.83 (19.98)
300 grains	7	136.35 (20.1)	27.54 (14.6)	106.0 (6.2)
1000 grains	8	135.96 (14.0)	27.15 (11.0)	100.0 (2.27)
>1000 grains	10	145.31 (12.7)	28.33 (7.8)	100.55 (2.53)

*The table is reprinted with permission from Ter-Avanesian (1978)*.

## Stress vs. Yield at High Temperature

**FIGURE 3:**
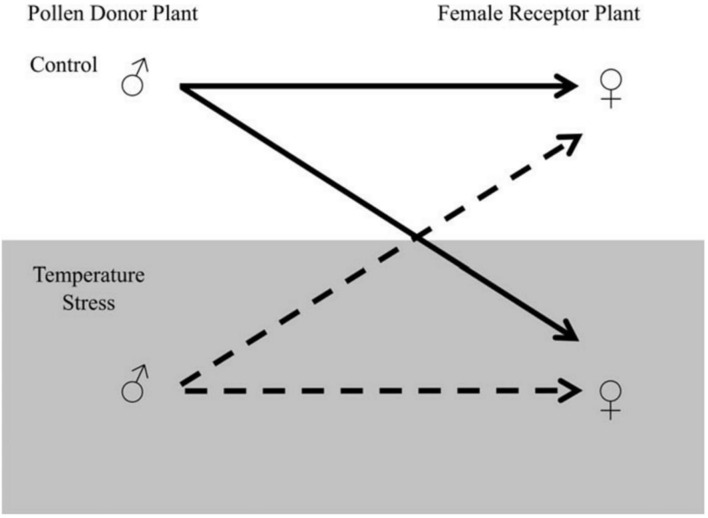
**Reciprocal crossing plan for temperature stress sensitivity**. The Pollen Donor Plant can be denoted by ♂ and the female receptor plant by ♀. The section at the bottom highlighted in gray represents temperature-stressed male and female contributions. Arrows illustrate the possible combination of crosses. The dotted line represents temperature-stressed male contributions and the solid line represents the control temperature male contribution. The figure is reprinted with permission from Zinn et al. (2010).

Final yield of cotton can be influenced by stress related to the elevated temperature. Negative correlation of high temperature was observed with cotton lint yield (Oosterhuis, 1999). Every year variations in the yield of cotton (a major anxiety for cotton producers) have been allied with impulsive differentiation in the temperature of growing seasons. The final lint yield was compared with mean maximum temperatures weekly after flowering during a study by Oosterhuis (1999). It was observed that major reduction in yield occurred when the average temperatures was more than 32°C. A high declining trend in fruiting efficiency has also been observed beyond 29°C (Reddy et al., 1996). When temperature exceeds above optimal range during the day, it declines the rate of photosynthesis and carbohydrate production. High temperature at night enhance the process of respiration and decrease the available carbohydrates (Loka and Oosterhuis, 2010), resulting in the reduction of various parameters (including boll size, seed setting, number of seeds/boll, and most importantly the quality and quantity of fiber; Arevalo et al., 2008). The major yield components of cotton are boll number and boll size. Boll retention reduced dramatically under extreme temperature and was observed to be highly sensitive component of cotton contributing to the final yield (Zhao et al., 2005). For instance, Reddy et al. (1991) found that 30°C day temperature followed by 20°C at night ensued the subsided capability to retain boll. This temperature causes high rate of squares abortion and young bolls. It is obvious from the literature that heat stress negatively influencing seed development and is one of the main aspects of yield reduction. Pettigrew (2008) suggested that minor variation in temperature under field conditions were not enough to cease the seed weight but this minor increase can cause a major reduction in seed number per boll. A simple breeding procedure can be followed to stop the degradation of yield in severe condition. A reciprocal crossing plan can be applied to conclude that the reproductive tissues are accountable for temperature stress sensitivity (**Figure 3**).

## Mechanism of Action to High Temperature Stress

### MicroRNAs Regulation

Abiotic stress imposes several deleterious effects at both cellular and molecular levels and also at proteins, and DNA levels (Yamaguchi-Shinozaki and Shinozaki, 2006). Usually abiotic stress results in abnormal cellular functions and an intensive and extensive molecular reprogramming at both the transcriptional and post-transcriptional levels. The master gene regulators are transcriptional factors which can regulate several genes at a time of stress and can respond to adverse condition (Yamaguchi-Shinozaki and Shinozaki, 2006).

It is interesting to know if the microRNAs (miRNA), post-transcriptional gene regulators are mostly involved in the undesirable regulation of transcriptional factors. Therefore, we described the role of miRNA in controlling certain key aspects at molecular level to withstand stress conditions of various types. Also, this information was utilized in joining certain broken links in the field of genetics and molecular breeding. First of all it is worth mentioning that miRNAs are known as ‘killer RNAs’ that are classified as endogenous non-coding small RNAs (Sun, 2012). This class of RNAs adversely affect almost all the metabolic and biological processes at the post-transcriptional level (Sun, 2012). The killer RNAs are of ∼21 nucleotides that are derived from single stranded RNA hairpin precursors. The nucleotides are cleaved by a double stranded specific ribonuclease (Dicer) in animals and Dicer-like1 (DCL1) in plants (Hutvagner et al., 2001; Reinhart et al., 2002; Tang et al., 2003). The recent development in high throughput sequencing technology helped in the identification of many low abundance non-conserved or species specific miRNAs in numerous plant species (Szittya et al., 2008; Pantaleo et al., 2010; Zhao et al., 2010; Song et al., 2011) with several classes.

Recent researches have revealed that several abiotic stresses (high temperature, drought, osmotic stress, and salinity) in different plant species (including rice, maize, *Arabidopsis*, populus, and even tobacco) can induce different expression of a particular set of miRNAs (Sun, 2012). Still, very little is known about the mechanism in cotton crop (Yin et al., 2012). In *Arabidopsis*, miR156, miR158, miR159, miR165, miR167, miR168, miR169, miR171, miR319, miR393, miR394, and miR396 have been reported for drought stress (Liu et al., 2009).

miR474 targets proline dehydrogenase (PDH) in maize has been currently observed to be up regulated in drought stress condition (Wei et al., 2009). Furthermore, 13 miRNAs have been nominated to be regulated differentially against drought stress: miR156, miR166, miR171, miR396, miR398, miR474, miR528, miR894, miR896, miR1432, miR1450, miR1867, and miR1881 in wild emmer wheat (Kantar et al., 2011).

Several miRNA families and their targets have been figured out by computational process based on the conserved characterization of miRNAs in cotton ovule and other developmental process (Abdurakhmonov et al., 2008; Sunkar and Jagadeeswaran, 2008). Whereas, the studies involved deep sequencing to validate the involvement of several miRNAs in the development of cotton (Zhang and Pan, 2009). Presently, 89 miRNAs are deposited at Plant Micro RNA database (PMRD) under *Gossypium*. Interestingly, several less conserved as well as highly conserved miRNAs families were observed to be specific to cotton tissues (Zhang and Pan, 2009). This process has been extensively studied in *Arabidopsis*, rice and other crops (Sunkar and Jagadeeswaran, 2008). But in cotton the role of miRNAs in abiotic stress tolerance is less understood and lacking considerable amount of literature (Yin et al., 2012). Therefore, a thorough understanding of cotton genome via advance high throughput sequencing technology is the demand of today’s scientific world. Such methodology should identify and classify any type of miRNA and elucidate their role in order to develop a super cotton germplasm. The identification of such miRNAs and its role in cotton will provide a breakthrough in genes identification related to abiotic stresses. Furthermore, the profiled miRNA and its link with stress regulatory networks can provide basis to manipulate the plants to with stand factors that can adversely affect the crop (Sunkar and Jagadeeswaran, 2008). Using this information a dominant cotton variety can be established to overcome the problem of high temperature and also withstand several other abiotic stresses.

In cotton, a set of miRNAs (miR2118, miR828, miR869, miR1030, miR159, miR165, miR170, miR319, miR529, and miR1884) has been reported that are up-regulated during high temperature stress. Identification of high stress responsive miRNAs in cotton has opened a new way for future molecular breeding. We can identify heat responsive miRNAs in cotton for different traits by using modern techniques. Overexpression of high temperature stress miRNAs in cotton will be helpful for the development of heat stress varieties. The plant hormone known as ABA is not only important for the extensive development, but adoption of plants to stress conditions (Cutler et al., 2010). The Phytohormone ABA can largely control the developmental stages of plants during stress condition through its regulation, production and several terms of signal transduction (Cutler et al., 2010). It is worth mentioning some key aspects of ABA to understand how it works in stress condition. This will help us to defend yield losses and understand its role in signal transduction under drought stress. ABA is formed through carotenoids from glyceraldehyde-3-phosphate and isopentenyl diphosphate in those cells containing plastids, i.e., in matures leaves and roots (Davies, 2010). ABA is one of the most important growth inhibitor that inhibits cell propagation by avoiding the loosening of cells before cell elongation (Zeevaart and Creelman, 1988). In addition, it plays a major role in plants’ signaling under drought stress. The rate of ABA synthesis during water stress conditions can be raised in root, which is then transported to the shoot and increased by 50% in leaf during water stress conditions (Christmann et al., 2005). Due to high concentrations of ABA in leaves the potassium and other related ions depart the guard cells and in response the stomata starts to close reducing the process of evaporation or water loss (Macrobbie, 1997). ABA also enhances the hydraulic conductivity of the root system and raises the root to shoot ratio in water deficit condition (Taiz and Zeiger, 2010) and can close the stomata to preserve the water source.

A “pleiotropic recessive *Arabidopsis* transposon insertion mutation,” hyl1 mutant was observed for the first time to evident the role of ABA-mediated responses and ABA hypersensitivity (Sunkar and Zhu, 2004). Different studies showed that ABA treatment regulated miR159 expression (Sunkar and Zhu, 2004). A seed specific transcription factor (TF) ABI3 helps the ABA to induce and accumulate miRNA 159 (Reyes and Chua, 2007). The induced miRNA 159 then cleaves other transcripts to up regulate the MYB33 and MYB101 causing the ABA signaling. Thus, miR159 has a central role to dilute the response of ABA in plants. The expression of miR393, miR397b, and miR402 can be up-regulated, while that of miR389a get down regulated, when treated the source plant with the ABA (Sunkar and Zhu, 2004). In the model *Arabidopsis* plant both miR160 (Liu et al., 2007) and miR417 (Jung and Kang, 2007) were noticed up-regulated, while miR169 and miR398 (Jia et al., 2009) got down-regulated with ABA treatment. Similarly, the expression of miR319 was discovered up regulated in ABA treated rice, On the other hand a down regulation of miR167 and miR169 was also spotted (Liu et al., 2009). In the ABA treated *Phaseolus vulgaris*, the induction of miR159.2, miR393, and miR2118 along with the up regulation of miR1514 and miR2119 were observed (Arenas-Huertero et al., 2009). The DNA methylation in *Physcomitrella patens* is also dependent on the level of PpbHLH-miR1026 regulon, which by itself rely on ABA (i.e., ABA application can promote miR1026 and inhibits PpbHLH target RNA; Khraiwesh et al., 2010). The MicroRNAs under ABA and high temperature stress conditions profiled using membrane array are given in **Table 3**. Understanding the role of ABA in detail and its molecular characteristic can unleash the most abstruse phenomenon of stress in cotton and in other crops as well. This mechanism can facilitate the scientific and breeding communities to manipulate the germplasm in desirable direction for the welfare of human beings (Ali and Yan, 2012). Therefore, the responsibility of scientist to increase yield of all crops are of prime importance for feeding the humanity and meet the demands of increasing world’s population.

**Table 3:** MicroRNA membrane array quantified data of stress treatments.

S. no	miRNA	Control	ABA	Heat
1	athmiR156a	1	1.22	2.95
2	athmiR157a	1	1.09	1.59
3	athmiR164a	1	0.56	3.74
4	ptcmiR164f	1	0.81	3.08
5	osamiR166l	1	1.04	1.93
6	ptcmiR166p	1	0.83	1.92
7	ptcmiR167h	1	1.58	2.06
8	athmiR168a	1	1.17	2.28
9	osamiR171b	1	2.15	2.69
10	zmamiR171c	1	1.39	3.66
11	zmamiR171f	1	2.23	2.71
12	athmiR171b	1	1.61	2.00
13	ptcmiR319i	1	0.81	4.18
14	athmiR390a	1	1.10	3.16
15	ptcmiR397b	1	3.01	4.76
16	athmiR398a	1	1.97	3.56
17	osamiR398b	1	1.81	3.42
18	osamiR399j	1	1.01	1.50
19	mtrmiR399d	1	1.40	3.52
20	ptcmiR399l	1	1.31	3.88
21	osamiR408	1	1.90	1.57
22	sofmiR408e	1	1.26	1.50

*The table is reprinted with permission from Jia et al. (2010)*.

MicroRNAs under ABA and high temperature stress conditions were profiled by using membrane array having controls. The data has been normalized by using the average of four membrane array external controls with ABA (100 μM-3 h) and Heat stress (45°C-1 h) treatments.

### Calcium Signals and High Temperature Stress

The signal transduction mechanism in plants is very complex and several other components are also involved in it apart from ABA. The most important one is calcium, having a strong feedback with key genes controlling the production of ABA (Murata et al., 2001). The effect of ABA on stomatal opening is indirectly linked through the induction of [Ca2+]cyt in guard cells, which means that [Ca2+]cyt can control the reduction in stomatal aperture. Moreover, the ABA induced Ca^2+^ in guard cells can play central role in signal transduction in order to regulate stomatal functions (Schmidt et al., 1995). The in depth study concerning the role of calcium in signal transduction will provide strong basis to understand the phenomenon at molecular level and to take necessary steps to minimize yield losses.

When cotton is exposed to high temperature stress as a result water availability to plants was decreased, promoting the drought conditions. Certainly, drought is among the major factors responsible for the plant stress leading to the inhibition of plant growth and development. Previous studies have provided enough evidences that feeding plants with exogenous Ca^2+^ can lead to high drought resistance, inhibiting the ROS generation, protecting the integrity cell membrane, regulates the photosynthetic reactions and production of plant hormone etc. (Qu et al., 2007). Additionally, the array of signal transduction in response to drought conditions by cellular Ca^2+^ not only regulate the physiological responses (Tuberosa et al., 2007), yet activate the major genes responsible to tolerate the adverse condition. Being a source of signal transduction, Ca^2+^ can also provide resistance to a set of abiotic stresses in plants. The whole process of the cellular Ca^2+^ signaling and transportation of Ca^2+^ is mainly controlled by a Ca^2+^ signals decoding proteins. It is well proven that the regulation of stomata in Ca^2+^ dependent scenario can induce [Ca2+]cyt at higher levels that instigates the loss in turgor by the surrounding guard cells.

Likewise, calcium is vital to have successful reproductive processes. The optimum concentration of calcium in the pistil and the pollen tube of several angiosperm species have promoted the normal growth of the tube from the stigma till ovule. Such promotion of the pollen tube in a well-organized manner ensures a successful fertilization (Qu et al., 2007). Also, the egg and sperm cells demand for small amount of calcium to endorse the fusion of gamete (Faure et al., 1994) and activation of egg (Digonnet et al., 1997).

### Role of HSPs

It is well-known that certain proteins accumulate in plant cells during high temperature stress to refrain them from the heat shock. The proteins are classified as HSPs that are extremely important to withstand adverse condition to give high yield. The rate of synthesis of these proteins augmented when plants were exposed to a high temperature in a natural habitat (Abrol and Ingram, 1996). Whenever, plant feels stress due to elevated temperature, a signal from the defense system initiates to synthesize higher levels of HSPs for protection. In a laboratory grown cotton experiment, the production and accrual of HSPs in cotton was noticed significantly high, when exposed to an elevated temperatures (38–41°C; Burke et al., 1985). From the genomic studies, it was also concluded that such expression of HSPs can be linked to temperature tolerance (Abrol and Ingram, 1996). Up to now the HSPs are classified in to three major classes on the basis of their molecular weights, i.e., HSP90, HSP70, and low molecular weight (LMW) proteins having *Mr* of 15–30 kDa. Each time, plants were forced to receive high temperature stress, produced and accumulated a very high quantities of LMW (200x), HSP70 (10x), and HSP90 (10x) to normal. The LMWs (mainly of 15–30, 70, and 90 kDa) in addition to its high quantities have the unique ability to be accumulated in plants during all the stages of development in response to high temperature. The role of HSPs to achieve heat tolerance, maintain cell integrity, avoid protein denaturation, and secure PSII became clear few decades back (Vierling, 1991). To date, less success has been achieved by over expressing the plant’s HSPs in order to adopt high temperatures. In the *Arabidopsis*, Lee et al. (1995) has successfully introduced HSP-reporter fusion genes in to plants and thus enhanced its tolerance to high temperature. The genes responsible for production of these proteins can be identified via different molecular approaches and there full information will facilitate the breeding community. Recently, a lot of markers have been explored in several cereals to identify the flanking markers of many adaptive traits. Especially, the single nucleotide polymorphism markers are abundant in plants like maize and rice. Several studies have shown that qualitative traits can be manipulated in desirable direction because numerous loci have been identified by genome wide association mapping (Kump et al., 2011).

### Role of Signaling Pathways during High Temperature Stress

It has been mentioned that plasma membrane (PM) acts as a primary heat sensor, detecting the stress condition via different approaches (Sangwan et al., 2002). These approaches involve the opening of calcium channels which lead to the calcium influx, and also may triggers a series of signaling pathways. The most important signaling pathways are calcium, kinases, reactive oxygen species (ROS), carbohydrate, TFs, gene expression regulation, and plant hormones etc. Triggered calcium signaling (TCS) can results in binding of calcium to calmodulin (CaM) and also activates the calcium-dependent protein kinases (CDPKs). Furthermore, the phosphatidylinositol-4-phosphate 5-kinases (PIPKs) can also be activated through TCS. The accumulation of PIPKs might trigger lipid signaling and CDPKs can activate mitogen-activated protein kinases (MAPKs; Sangwan et al., 2002). Calcium signaling also can activate the production of ROS by activation of respiratory burst oxidase homolog D (RBOHD) in PM (Miller et al., 2007). The accumulation of ROS could activate the antioxidant defense system [peroxidase (POD); catalase (CAT); superoxide dismutase (SOD)] mediating by ABA signaling. As a result ROS, activated MAPKs and heat shock factors (HSFs), and could enhance the high temperature stress response, which can lead to programmed cell death (PCD; Blanvillain et al., 2011). Furthermore, the CDPKs, PIPKs, and MAPKs can also be involved in kinases signaling. In kinases signaling, casein kinase I (CKI), which can affect the calcium signaling, ABA, ROS, and carbohydrate signaling through interaction with starch synthases cause male sterility under high temperature (Min et al., 2013). SNF1-related protein kinase (SnRK) could be a key enzyme in carbohydrate signaling and performing a key role in sugar signaling and ABA signaling (Rolland et al., 2006). Different sugars (sucrose, glucose, fructose, trehalose, starch, and so on) may be involved in high temperature stress responses by mediating energy metabolites through molecular mechanism. Cytosol unfolded protein (Cyt-UPR) and endoplasmic reticulum unfolded protein (ER-UPR) function through TFs, such as HSFs, bZIPs, WRKY, DREB, ARF, JAZ, and ERF (Chen et al., 2012; Scharf et al., 2012). HSFs, known as key transcriptional regulators of basal high temperature stress response are playing a very important role in Cyt-UPR pathways. The bZIPs release from the ER membrane and is involved in ER-UPR signaling pathway. Other TFs mainly involved in plant hormones (such as IAA, JA, ABA, ET, BR, and SA) signaling pathways (Sakata et al., 2010), yet some occcupied central role in expressions of genes to regulate the pathway. However, DNA methylation, histone constitution and modification, and microRNA are referred to as the epigenome. The regulation of epigenome is a method for plant rapid adaptation to high temperature stress. Plants respond to changes in the ambient temperature through a series of complex reactions (such as, accumulation of the HSPs, protein homeostasis, and metabolome homeostasis) that can cause oxidative damage and leads to PCD.

### Epigenetical Regulation and Elevated Temperature Stress

Histone modifications through histone tails occurred at different amino acids. These modifications are achieved through histone methylation, acetylation, ubiquitination, and phosphorylation. These histone modifications play crucial role in the transcriptional regulation of genes through opening or closing of chromatin (Li et al., 2007). H3K4me and H3K36me are involved in the opening of chromatin and increase transcriptional regulation of genes, while H3K9me and H3K27me are involved in closing of chromatin and decrease the transcriptional regulation of genes. The key role of histone modifications on transcriptional regulation of gene, genome management and plant development have been thoroughly studies over the years (Liu et al., 2010; He et al., 2011). The modifications in histone proteins in various plant species can be affected by rise in temperature. The effect of high temperature at genetic level in transmission vector (*Chlamydomonas reinhardtii*) revealed a significant rise in acetylated H3/H4 histone with decrease in H3K4me1 histone (Strenkert et al., 2011).

According to Strenkert et al. (2011), the acetylation of histone H3/H4 can be mediated by a transcriptional factor (HSF1) after heat shock. However, the level of acetylated H3 histone in the forest tree, cork oak was dipped with rise in temperature (Correia et al., 2013). The deacetylated H3 further involves in the repression of chromatin in the promoter region of the gene and thus leads to failure in gene transcription. The modification in histone proteins at high temperature is equally important in the development of rice seeds and cotton anthers. The exposure of rice seed to high temperature during developmental stage can bring changes in the regulation of *OsFIE1* (Folsom et al., 2014), where in anthers of cotton, three genes get down regulated (including 2-jumonji C and a histone monoubiquitnation; Min et al., 2014). A strong association is required between enhancers and transcriptional factors to increase the transcription of genes. This association will lead to histone modifications and opening of chromatin as a result gene transcription will be increased. Therefore, it is vital to understand the role of enhancers in gene transcription and find out way to withstand adverse environmental condition.

Genomic elements named as enhancer is nonetheless underestimated for determination of gene expression. Enhancers contain short sequence motifs. These short motifs are used as binding sites for the transcriptional factors. However, how enhancers sequences and short motifs of TFs are correlated to enhancer activity are not clear, and sequences requirement for enhancer activity is still unknown (Yáñez-Cuna et al., 2014). Understanding this phenomenon to the best of its level needs sequences requirement for enhancer activity and how enhancers play role in gene expression. On the other hand, we have to figure out that how association between transcriptional factors and enhancers play key role in the opening of chromatin.

Latest developed method formaldehyde-assisted isolation of regulatory elements (FAIREs) and DNase1 sensitivity has exposed that active enhancers exhibit accessible DNA regions. After applying high-throughput sequencing together with genome-wide, FAIRE-seq and DNase-seq give a snapshot of genomic regions of high physical accessibility, including enhancers and promoters (Giresi and Lieb, 2009). Active enhancers possess DNA accessible regions flanked by H3K4me1 and H3K27ac peaks. Moreover, active enhancers also contain peak for the recruitment of coactivator p300. Conversely, the inactive enhancers exhibit repressive marks H3K27me3 or H3K9me3 (Mikkelsen et al., 2007). Latest study has reported that enrichment of dinucleotides repeat motifs (DRMs) increase the activity of enhancers; especially in those enhancers which are broadly active across different cell types. Yáñez-Cuna et al. (2014) validated the transcriptional factors motifs and DRMs. We need to unleash the major role of transcriptional factors in maintenance and establishment of chromatin structure to better understand the gene transcription. A specific group of TFs named as a pioneer TFs can change the fate of the cells by interfering with the sites in silent chromatin (Iwafuchi-Doi and Zaret, 2014). Pioneer TFs have a role in reprogramming one cell into another and possess the ability to recognize and engage lineage-specific genes that are developmentally silenced. Developmentally silenced genes are present in closed chromatin structure that contains repressive histone modification mark (Iwafuchi-Doi and Zaret, 2014).

### Non-coding RNAs and Elevated Temperature Stress

Enhancer plays pivotal role in gene regulation but function of enhancer transcript in regulatory system is unknown. Latest research has exposed that transcriptional enhancers produce non-coding RNAs known as eRNAs. eRNA has a central role to initiate or activate transcription either through stabilize binding or proximal promoter of target genes (Pnueli et al., 2015). The transcription of eRNA at enhancer state can improves gene expression, which is specific to the tissue. So, the presence of certain eRNAs in a specific tissue and its correlation with the expression of target genes suggests that eRNAs have gene activation potential. Furthermore, it has been found that most of the regions in eRNAs has sequences with secondary structures that are similar to those of microRNAs (Pnueli et al., 2015).

Currently, an epigenetic pathway in response to temperature related stress has unleashed the active participation of non-coding RNA (Li et al., 2014). This was evident, when a non-coding RNA known as transacting siRNA precursor 1 (TAS1) was changed to a double stranded RNA via RDR6 under the influence of miR173. The produced double stranded RNA was converted in to 21-nucleotide, transacting interfering RNAs (Ta-siRNAs) by using Dicer like 4 (DCL4) RNase III enzyme (Allen et al., 2005). The siRNAs that was derived from TAS1 can be provoked by heat that can bind to HEATINDUCED TASI TARGET (HTT) genes. HsFA1 transcriptional factor has the ability to bind HSE directly in the HTT promoter region and stimulates thermotolerancer (Li et al., 2014).

It was quite intriguing that small RNAs antagonistically suppress the Hsf1A targets, ONSEN retrotransposons and HTT genes under ambient temperatures. Transcriptional factors of small RNAs and Hsf1A may handle the responses from high temperature stress via regulating the target genes. On the other hand, during repeated water stress conditions, the pathway of minor RNA’s can act as a stress recall. The accumulation of H3K4me and occupancy of RNA pol II under rehydration process during drought recovery (Ding et al., 2012) depicts the deposition of minor RNAs. This accumulation of minor RNAs mainly targets the HTT or ONSEN can be overdue during the salvage after high temperatures shock. The above mentioned findings collectively, suggest the identification of pioneer transcriptional factors in cotton. These pioneer TFs not only open the close chromatin but they will also increase the enhancer activity and as a result high enhancer transcript will be produce which will increase the expression of target genes.

## Futuristic Perspective

Production of an ideotype in term of quality, quantity, and resistance is a cumbersome task in breeding. Even with the advent of modern biological techniques this task cannot be accomplished successfully to improve the living standards of humanity. The role of conventional breeding in developed countries is continuously on decline, while in developing countries most of the time, new technology and approaches are lacking because of low funds and lack of new technology. Therefore, sharing the information, technology and germplasms resources can solve many issues in poor and developing countries. On the other hand, scientists need to think about the potential threat of increase in annual temperature and take necessary action in proper direction that can lead to increase the resilience of each crop to withstand malevolent conditions. Some of the changes in climatic factors can have alarming effects on crop production for instance a slight increase in temperature (which has already offset a significant portion of yield increase arisen from various factors) can drastically affect yield and adversely influence the agronomic traits (Shah et al., 2014). For cotton crop, its production has been seriously affected by both abiotic and biotic stresses. High temperature and drought are two important stresses among the abiotic factors, which cause a huge loss of cotton yield each year. Considering this whole scenario it is extremely difficult to predict the future of cotton area, production and consumption. Total area of cotton will depend on competition of cotton with other crops and the availability of irrigation water. The forecasted consumption will rise up to approximately 49 million tons by 2025. As a result 65.5 million ha will be required to fulfill the demands of cotton at current yield capacity. Establishing new cotton production zones, particularly in non-irrigated areas, will depend on increasing the irrigation water and boost the water use efficiency. It seems a bit difficult and almost impossible to increase the irrigation water but an alternative way of high yielding, disease resistance and stress tolerance varieties can meet consumption by 2025. If the cultivated area remains the same but we increase the yield to 1,000 kg/ha, 25% productivity can be achieved.

Another possibility may be identifying the potential gene regulatory networks which may come through genome wide data for high temperature stress. This will be a crucial improvement in the era of green biotechnology in relation to genomic selection, epigenomics and marker assisted selection. Advance regulatory components inside these networks could be sorted out as main pillars or as key candidate genes via system biology to exploit their functional relevance. System Biology approaches can leads scientists to their targets in a scientific manner. Therefore, embracing the approach of system biology and combining of wet-lab experiments with new bioinformatics tools several goals can be accomplished at a time. For example functional study of key genes, miRNAs, transcriptional factors, Phytohormones, protein–protein interaction, miRNAs and protein interaction can be identified at network level.

**FIGURE 4:**
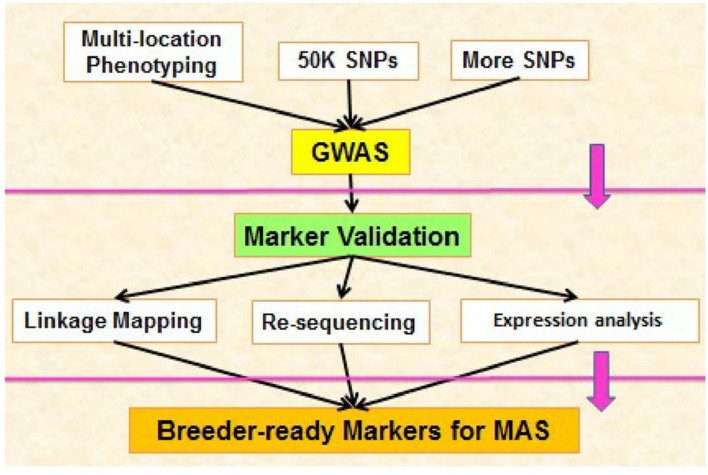
**Process of developing markers via Genome wide association studies**.

Future study should be focus on above mentioned regulatory components, first we should identify novel regulatory components in cotton and then we should elucidate how these components operate alone and how they interact with other regulatory components during high temperature stress. Studying of molecular interaction among these regulatory components will provide us deep knowledge for making different strategies against heat stress. Candidate genes in cotton against high temperature stress can be identified through GWAS. These genes should be induced in different parts of cotton during heat stress. For example pollen grains are highly sensitive against high temperature stress, so scientists should focus on this aspect during identification and transformation of genes in cotton against heat stress. Most dominant procedure of GWAS/ MAS for identification of loci related to adverse condition can be followed for secure future (**Figure 4**).

We have mentioned the role miRNAs against different abiotic stresses in plants. Recently, some miRNAs have been identified in cotton, they play important role during heat stress. There is need to find out new miRNAs in cotton which should have strong interaction with genes. System biology approaches has explored that each miRNA can have several targets and that each single gene can be regulated by several miRNAs which showed that the design of study must not involve “one miRNA vs. one target.” This concept is likely to wrong because one miRNA may have several target genes. As a result, in the light of system biology we can identify those miRNAs which have multiple functions during high temperature stress. According to this research that miRNAs have multiple functions, dual benefits can be obtained at a time. Cloning of those genes which can be regulated by multiple miRNAs during heat stress will prove reinforce against harmful environmental conditions. In the same way, overexpression of those miRNAs in cotton which can induce multiple genes will improve the performance of cotton plant against high temperature stress. By adopting multiple strategies, we can produce those cotton varieties which can withstand against abiotic stresses.

Transcription factors consists of a group/s of key regulatory genes that can play an important roles in the mechanism of stress-responsive genes. Over-expression of a single TF can trigger a group of target loci that function in a systematic approach to respond adverse environmental condition. Therefore, genetic engineering of TFs will be a powerful approach to improve the genetic ability of crop plants against abiotic stresses. Importance of identification and overexpression of new TFs in cotton can’t be ignored. TFs also play pivotal role during signaling pathways against high temperature stress. We should also focus on those TFs which have powerful connection with some miRNAs and candidate genes during high temperature stress in cotton. Overexpression of TFs in cotton will also be proved powerful strategy for the development of heat resistant cotton varieties.

Phytohormones also play major roles during signaling pathways against high temperature stress. Recently, in anther of cotton, it has been observed that expression pattern of different genes is changed in different Phytohormones signaling pathways in stress condition. It has been observed that the loci involved in auxin metabolism and signaling comprise the biggest group, whereas jasmonic acid, cytokinin, and brassinosteroid signaling pathways are also inflated by high temperature stress. This study indicates a complicated cross-talk among Phytohormones which can be involved in the development of cotton anther during high temperature stress (Min et al., 2014). Strong understanding of dynamic modifications in the cross-talk among TFs, Phytohormones, and genes in reaction of heat stress and their significance for best possible plant development is an important challenge. Keeping in view this important factor, the phenomenon of systems biology will be extremely helpful for the identification of cross-talk among Phytohormones, TFs, candidate genes, and miRNAs. Scientist should study the molecular mechanism and pathway of above regulatory components for better understanding cross-talk among Phytohormones, TFs, candidate genes and miRNAs. Overexpression of those candidate genes in cotton which can play major role of signal transduction among Phytohormones, TFs and miRNAs will be proved powerful tool for the development of heat resistant varieties.

## Author Contributions

KZ, FA, MY, and DS developed the concept of this task; FA, KZ, WH, ZA, and TS contributed the drafting of the article; FS, WW, AI, CQ, and FA helped in drafting and critically revising the manuscript.

## Conflict of Interest Statement

The authors declare that the research was conducted in the absence of any commercial or financial relationships that could be construed as a potential conflict of interest.
